# Specific protein homeostatic functions of small heat‐shock proteins increase lifespan

**DOI:** 10.1111/acel.12422

**Published:** 2015-12-25

**Authors:** Michel J. Vos, Serena Carra, Bart Kanon, Floris Bosveld, Karin Klauke, Ody C. M. Sibon, Harm H. Kampinga

**Affiliations:** ^1^ Department of Cell BiologyUniversity Medical Center GroningenUniversity of GroningenGroningenThe Netherlands; ^2^ Laboratory of Endocrinology and RadiochemistryDepartment of Clinical ChemistryAcademic Medical CenterAmsterdamThe Netherlands; ^3^ Dipartimento di Scienze BiomedicheMetaboliche e NeuroscienzeUniversita' di Modena e Reggio Emiliavia G. Campi 28741125ModenaItaly; ^4^ Polarity Division and Morphogenesis TeamInstitut CurieCNRS UMR 3215, INSERM U93426 rue d'Ulm75248Paris Cedex 05France

**Keywords:** protein homeostasis, longevity, aging, *Drosophila melanogaster*, small heat‐shock protein, HSPB family

## Abstract

During aging, oxidized, misfolded, and aggregated proteins accumulate in cells, while the capacity to deal with protein damage declines severely. To cope with the toxicity of damaged proteins, cells rely on protein quality control networks, in particular proteins belonging to the family of heat‐shock proteins (HSPs). As safeguards of the cellular proteome, HSPs assist in protein folding and prevent accumulation of damaged, misfolded proteins. Here, we compared the capacity of all *Drosophila melanogaster* small HSP family members for their ability to assist in refolding stress‐denatured substrates and/or to prevent aggregation of disease‐associated misfolded proteins. We identified CG14207 as a novel and potent small HSP member that exclusively assisted in HSP70‐dependent refolding of stress‐denatured proteins. Furthermore, we report that HSP67BC, which has no role in protein refolding, was the most effective small HSP preventing toxic protein aggregation in an HSP70‐independent manner. Importantly, overexpression of both CG14207 and HSP67BC in *Drosophila* leads to a mild increase in lifespan, demonstrating that increased levels of functionally diverse small HSPs can promote longevity *in vivo*.

## Introduction

The imbalance in overall protein homeostasis is a crucial factor in the development of heritable age‐related neurodegenerative diseases and during normal aging (Dobson, 2003; Vacher *et al*., 2005; Zhang *et al*., 2005; Arslan *et al*., 2006; Haass & Selkoe, 2007; Morimoto, 2008). Achieving and maintaining the correct three‐dimensional protein structure is a continuous struggle within cells. Firstly, folding of proteins toward an active biological state is challenged by the crowded environment within the cell, which may lead to off‐pathway reactions resulting in protein aggregation (Ellis & Minton, 2006; Engel *et al*., 2008; Homouz *et al*., 2008). Protein misfolding can further originate from direct protein damage (e.g., oxidation, thermal denaturation), but can also originate from age‐related mutations, molecular misreading (van Leeuwen *et al*., 2000), splicing errors (Pettigrew & Brown, 2008), or errors in translation (Parker, 1989; Kramer & Farabaugh, 2007). While cells are challenged by an accumulation of oxidized, misfolded, and aggregation‐prone proteins, their capacity to deal with accumulated protein damage declines with aging (Liu *et al*., 1989; Bulteau *et al*., 2002; Ferrington *et al*., 2005; Naidoo *et al*., 2008).

As molecular chaperones, heat‐shock proteins (HSPs) play a central role in protein homeostasis: They safeguard protein conformation and folding and assist in the assembly and disassembly of protein complexes, and in protein degradation. By their ability to bind non‐native polypeptides, they maintain their substrates in a state competent for subsequent folding or, when folding is not successful, for degradation by the ubiquitin–proteasome system (Urushitani *et al*., 2004) or through autophagy (Carra *et al*., 2008). Hereby chaperones can prevent toxic protein aggregation, and as such, they have been implicated as protectors against age‐related protein folding diseases (Rujano & Kampinga, 2008) and as supporters of healthy aging (Hsu *et al*., 2003; Morrow & Tanguay, 2003; Walker & Lithgow, 2003; Morley & Morimoto, 2004; Morimoto, 2008). Indeed, activation of all stress‐inducible HSPs, either by overexpression of the heat‐shock factor‐1 (HSF‐1) (Morley & Morimoto, 2004; Morimoto, 2008) or via caloric restriction and the accompanying insulin signaling (Hsu *et al*., 2003), was shown to delay the onset of protein folding diseases and to induce longevity in otherwise healthy animals.

Interestingly, even the sole overexpression of single members of the small HSP family was shown to support longevity both in *Caenorhabditis elegans* (Walker & Lithgow, 2003) and in *Drosophila melanogaster* (Aigaki *et al*., 2002; Morrow & Tanguay, 2003; Morrow *et al*., 2004; Wang *et al*., 2004). The small HSPs tested so far (HSP22, HSP23, HSP26, HSP27) share the capacity to facilitate refolding of stress‐denatured substrates *in vitro* (Morrow *et al*., 2006), supporting the hypothesis that maintenance of global protein homeostasis is essential for longevity. To elucidate which *Drosophila* small HSPs might be the most potent in preventing protein misfolding or the toxic aggregation of protein damage upon aging, we cloned all members of the *Drosophila* small HSP family (excluding the mitochondrial HSP22) and compared their ability to assist in refolding of stress‐denatured substrates and/or in preventing aggregation of disease‐associated misfolded proteins in living cells. We identified CG14207 as novel and potent small HSP member that exclusively assisted in HSP70‐dependent refolding of stress‐denatured proteins. Furthermore, we report that HSP67BC, which has no role in protein refolding, was the most efficient small HSP preventing toxic protein aggregation in an HSP70‐independent manner. Importantly, overexpression of both CG14207 and HSP67BC in *Drosophila* leads to increased lifespan, implicating that increased levels of these small HSPs can prevent aging *in vivo*.

## Results

### Heat inducibility of the *Drosophila* small HSPs

**Figure 1:**
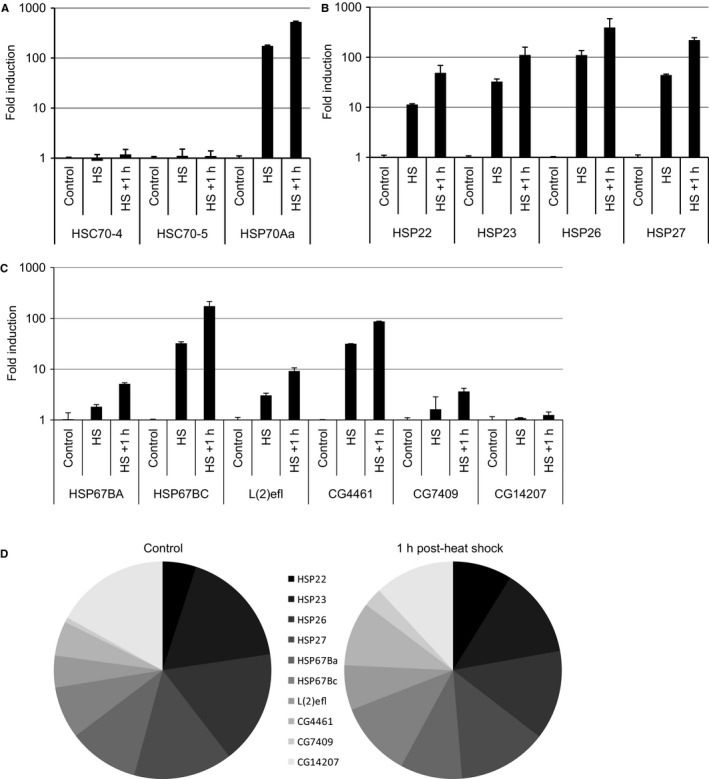
*Drosophila melanogaster* small heat‐shock proteins (HSP) family and heat inducibility. Transcript levels of HSC70‐4, HSC70‐5, HSP70Aa (A), the classical *D. melanogaster* small HSP genes (B), and the nonclassical small HSP members (C) were analyzed directly after heat shock (30 min at 38 °C) and 1 h after heat shock using quantitative RT–PCR. Relative mRNA abundance before and after heat shock is depicted in panel D. (CG13133 mRNA was not detected in S2 cells.)

To identify all *Drosophila* small HSPs, we first employed a comprehensive *in silico* approach and identified 11 candidates (see Experimental procedures). Certain members of all major HSP families are known to be induced upon proteotoxic stresses, including heat shock and exposure to heavy metals (Mosser *et al*., 1988). To analyze which of the 11 *D. melanogaster* small HSPs are heat‐inducible, we heat‐shocked *Drosophila* S2 cells at 38 °C for 30 min and analyzed the small HSP mRNA levels by qPCR. As controls, we measured the mRNA levels of HSC70‐4 and HSC70‐5, two constitutively expressed genes not responsive to heat shock (Ashburner & Bonner, 1979; Ish‐Horowicz *et al*., 1979), and HSP70Aa, a highly heat‐inducible gene (Fig. 1A). The four classical small HSPs (HSP22, HSP23, HSP26, and HSP27) were all highly induced after a heat shock (Fig. 1B), consistent with previous findings (Marin *et al*., 1996; Michaud *et al*., 1997). Apart from CG14207 and CG13133, all the other small HSPs were also found to be heat‐inducible (Fig. 1C). Expression of CG13133 was below the detection limit at both control and heat‐shock conditions. The most strongly induced nonclassical members were HSP67BC and CG4461, while HSP67BA, L(2)EFL, and CG7409 were moderately induced (Fig. 1C).

qPCR data were subsequently used to estimate the relative contribution of each member to the total pool of small HSP mRNA before and after heat shock. In S2 cells, the most abundantly constitutively expressed small HSPs are HSP23 (17.6% of the total pool), HSP26 (16.9%), HSP27 (14.7%), and CG14207 (17%) (Fig. 1D). After a heat shock and a recovery period of 1 h, the pool of small HSP mRNA has shifted toward a more homogenous distribution with comparable levels of HSP23, HSP26, HSP27, HSP67BC, HSP67BA, and CG4461 (Fig. 1D).

### CG14207 and CG7409 are the most active small HSP members in assisting refolding of heat‐denatured luciferase

**Figure 2:**
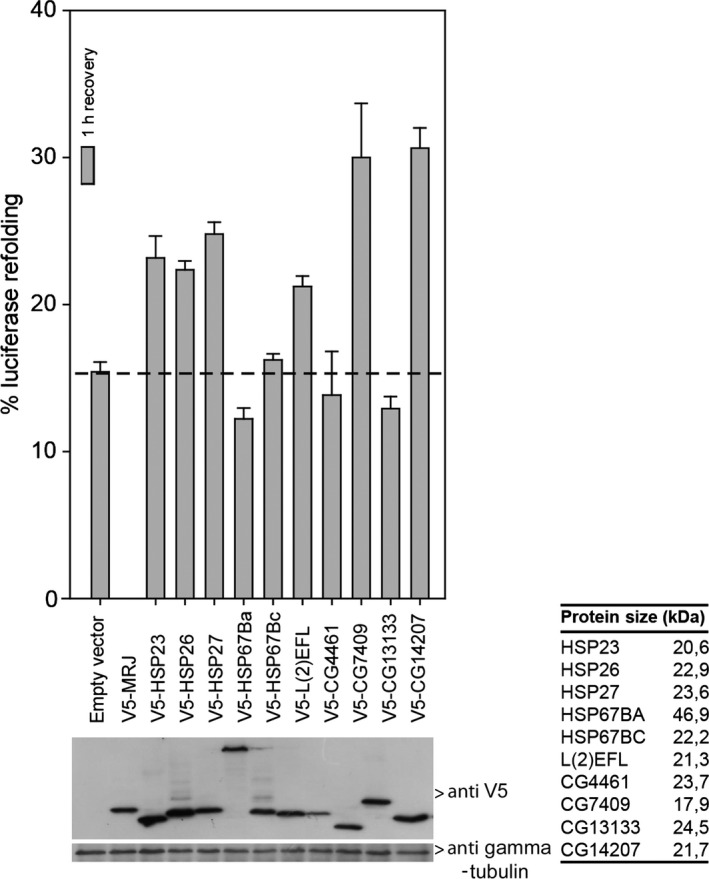
Effect of overexpression of the *Drosophila melanogaster* small HSP family in S2 cells on refolding heat‐inactivated firefly luciferase. *Drosophila melanogaster* small HSPs and firefly luciferase were coexpressed in Schneider's S2 cells. Twenty‐four hours after transfection, S2 cells were heated for 30 min at 38 °C reducing luciferase activity to < 5%. Next, cells were re‐incubated for 1 h at 25 °C to allow for (chaperone‐assisted) luciferase refolding. Luciferase activities are plotted relative to the activity in unheated cells (=100%). Data are mean ± SD of three independent experiments. Lower panel shows expression of chaperones using anti‐V5 antibodies.

It has been shown for several small HSPs that they can maintain substrates in a folding competent form both *in vitro* and *in vivo* (Mogk *et al*., 2003; Cashikar *et al*., 2005). *In vitro*, the addition of the HSP70/HSP40 refolding machinery is required for the refolding reaction (Lee *et al*., 1997). This has been reproduced in living cells using luciferase as a substrate (Nollen *et al*., 2001; Bryantsev *et al*., 2007). Here, we tailored the cellular luciferase refolding assay for *Drosophila* S2 cells and characterized which of the *Drosophila* small HSPs could enhance luciferase refolding upon overexpression. The mitochondrial HSP22 was excluded from our analyses as our cellular assays were only tailored for the cytosolic and nuclear compartments. We achieved relatively comparable expression levels for all small HSPs (Fig. 2, bottom) although expression of HSP23 and HSP26 was somewhat higher and expression of CG4461 was somewhat lower than that of the other seven sHSPs that showed similar expression levels. Consistent with *in vitro* data (Morrow *et al*., 2006), overexpression of the classical small HSPs (HSP23, HSP26, and HSP27) increased luciferase refolding (Fig. 2, top). Although less efficient, overexpression of L(2)EFL also led to improved luciferase refolding, whereas HSP67BA, HSP67BC, CG4461, and CG13133 had no effect. Interestingly, overexpression of CG7409 and the non‐heat‐shock‐inducible CG14207 resulted in the highest level of refolding 1 h after heat shock (Fig. 2, top).

**Figure 3:**
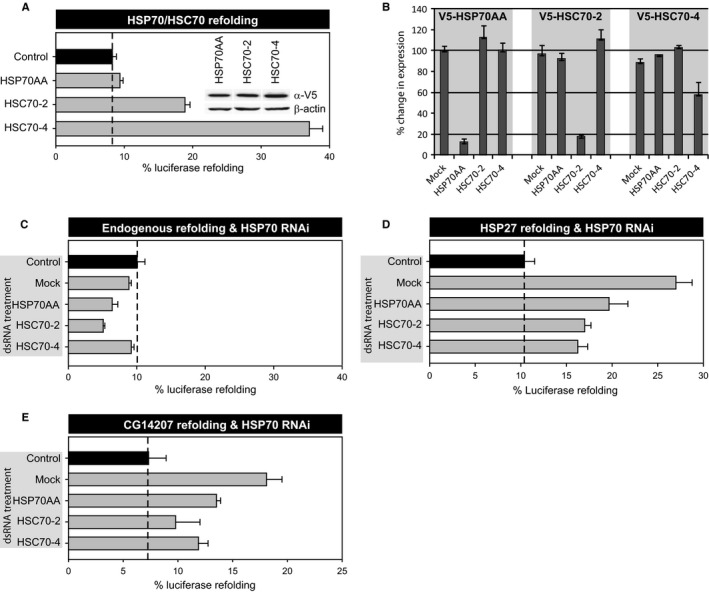
Dependency of HSP27‐ or CG14207‐assisted luciferase refolding on HSP70 (A) Firefly luciferase was coexpressed in Schneider's S2 cells together with eGFP (control) or *Drosophila melanogaster* HSP70s. Twenty‐four hours after transfection, S2 cells were heated for 30 min at 38 °C reducing luciferase activity to < 5%. Next, cells were re‐incubated for 1 h at 25 °C to allow for (chaperone‐assisted) luciferase refolding. (B–D) Schneider's S2 cells were transfected with dsRNA targeting individual HSP70s, and first RNAi efficiency and specificity were tested. Hereto, the various dsRNAs were coexpressed with V5‐HSP70AA, V5‐HSC70‐2, or V5‐HSC70‐4, and their levels were quantified from Western blots and expressed relative to expression without the dsRNA (B). Next, luciferase refolding was determined in the presence of dsRNA targeting endogenous HSP70AA, HSC70‐2, or HSC70‐4 alone (C) or combined with coexpression of V5‐HSP27 (D) or V5‐CG14207 (E), and luciferase refolding was determined as under panel A. In all panels, luciferase activities are plotted relative to the activity in unheated cells (=100%). Data are mean ± SD of three independent experiments.

To analyze whether *D. melanogaster* small HSPs, like bacterial, plant, and mammalian small HSPs (Lee & Vierling, 2000; Mogk *et al*., 2003; Bryantsev *et al*., 2007), also require HSP70 machines for refolding, we first tested which of the *D. melanogaster* HSP70s could promote refolding of heat‐denatured luciferase. Hereto, we cloned a selection of the *D. melanogaster* HSP70 family (Table S2, Supporting information) and analyzed their effect on luciferase refolding. Whereas all HSP70 proteins were expressed at equal levels, overexpression of both *D. melanogaster* HSC70‐2 and HSC70‐4, but not HSP70AA, enhanced luciferase refolding in S2 cells (Fig. 3A). This suggests either that HSP70AA lacks the ability to assist in refolding heat‐denatured luciferase or that specific cofactors that are required for its activity are rate‐limiting under these conditions. Subsequently, we downregulated the individual *D. melanogaster* HSP70s using dsRNA molecules to assess their role in protein refolding (Table S3, Supporting information). As no available antibodies can distinguish HSP70AA, HSC70‐2, and HSC70‐4, we tested the specificity and effectiveness of the dsRNAs using overexpression of V5‐tagged HSP70AA, HSC70‐2, or HSC70‐4. All dsRNA constructs were specific and significantly downregulated the expression of the targeted V5‐tagged protein (Fig. 3B). Indeed, the luciferase refolding capacity was substantially inhibited upon HSC70‐2 downregulation (Fig. 3C). dsRNA‐mediated knockdown of HSC70‐4 was less effective in downregulating HSC70‐4 and induced no significant effect on refolding (Fig. 3C). In contrast, dsRNA directed against HSP70AA, which did not enhance luciferase refolding upon overexpression, reduced refolding, suggesting that endogenous HSC70AA does play a role in refolding of heat‐denatured luciferase under physiological conditions. Next, we combined overexpression of V5‐HSP27 (Fig. 3D) and V5‐CG14207 (Fig. 3E) with downregulation of HSP70 members to assess the role of HSP70 proteins in sHSP‐mediated refolding. Refolding of luciferase in the presence of both V5‐HSP27 and V5‐CG14207 was considerably reduced by downregulating HSP70AA, HSC70‐2, or HSC70‐4 (Fig. 3D,E). Thus, our results strongly suggest that the refolding capacity of *D. melanogaster* HSP27 and CG14207 is partially dependent on an intact HSP70 machine.

### HSP67BC is the most potent suppressor of polyglutamine aggregation

**Figure 4:**
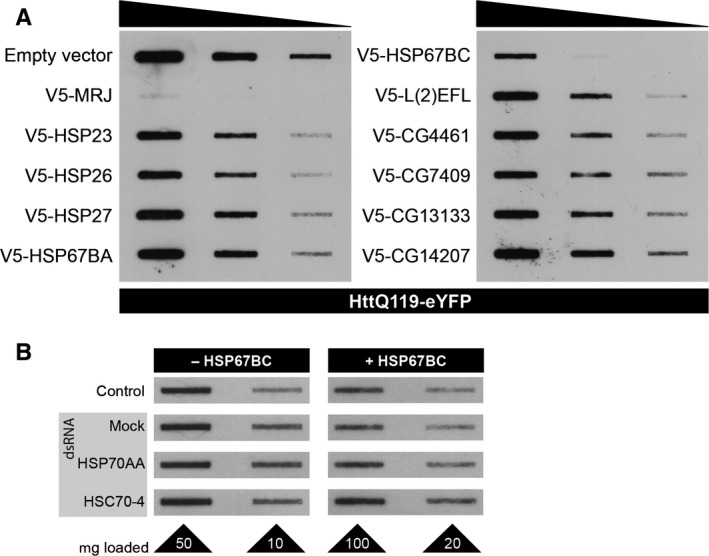
Effect of overexpression of the *Drosophila melanogaster* small heat‐shock proteins (HSP) in S2 cells on preventing aggregation of polyQ proteins. (A) EGFP‐Htt‐Q119, harboring exon 1 fragment of the huntingtin protein containing 119 glutamine repeats, was coexpressed in Schneider's S2 cells together with an empty vector (control) or the various *D. melanogaster* small HSPs. MRJ, a well‐known potent inhibitor of polyQ aggregation (Chuang *et al*., 2002; Fayazi *et al*., 2006), was included as a positive control. Forty‐eight hours after transfection, S2 cells were lysed and analyzed using the filter trap assay. Serial fivefold dilutions were loaded on cellulose acetate membranes and probed with anti‐GFP antibody. (B) EGFP‐Htt‐Q119 was coexpressed in Schneider's S2 cells without (−) or with (+) HSP67BC together with dsRNA targeting *D. melanogaster* HSP70Aa or HSC70‐4. PolyQ aggregates were detected using the filter trap assay using two dilutions. Samples coexpressing HSP67BC were loaded at higher concentrations to better visualize trapped aggregation.

Having identified CG14207 as the most potent *Drosophila* small HSP promoting HSP70‐dependent protein refolding, we next investigated which of the *Drosophila* small HSPs was the most effective in preventing toxic protein aggregate formation. Several protein folding diseases are characterized by the formation of toxic aggregates such as Alzheimer's disease, Huntington's disease, and amyotrophic lateral sclerosis. Some small HSPs have been reported to suppress the aggregation of such disease‐related proteins (Wilhelmus *et al*., 2006; Carra *et al*., 2008), and we have found that this is not always related to their capacity to refold heat‐denatured luciferase (Vos *et al*., 2010). Therefore, we tested which of the *Drosophila* small HSPs could suppress aggregation of an EGFP‐tagged huntingtin exon‐1 containing 119 glutamines (EGFP‐HDQ119) in S2 cells. The Dm ortholog of the human DNAJB6, MRJ, previously identified in a screen for suppressors of polyglutamine (polyQ) toxicity (Fayazi *et al*., 2006; Hageman *et al*., 2010) was used as a positive control and indeed completely inhibited aggregate formation in S2 cells as demonstrated using filter trap binding (Fig. 4A). Most small HSPs showed only minor or no effects on polyQ aggregate formation. However, overexpression of HSP67BC was very effective in preventing polyQ aggregation (Fig. 4A). Interestingly, this small HSP did not support luciferase refolding in *Drosophila* S2 cells (Fig. 2) and was identified as the ortholog of the human HSPB8 that in conjunction with BAG3 can reduce polyQ aggregation in mammalian cells (Carra *et al*., 2010).

Next, we tested whether HSP67BC requires a functional HSP70 machine to prevent polyQ aggregation. RNAi against HSP70AA and HSC70‐4 that resulted in the inhibition of the refolding promoting activity of CG14207 (Fig. 3E) did not lead to an increase in polyQ aggregation (Fig. 4B), indicating that the protective effect of HSP67BC against polyQ aggregation does not require HSP70 activity.

Combined, our findings demonstrate that the *Drosophila* small HSPs have versatile functions and that HSP70‐independent prevention of protein aggregation (HSP67BC) and HSP70‐dependent stimulation of protein refolding (CG14207) can be separated into two different proteins.

### *In vivo* effects of HSP67BC on polyQ toxicity

To determine whether the observed protective effect of HSP67BC on polyQ aggregation could be extended to an *in vivo* setting, we employed the ataxin‐3 (SCA3) fly model (Bilen & Bonini, 2007). This fly model expresses the ataxin‐3 gene with 78 CAG repeats under the control of the *UAS*/*gmr‐GAL4* expression system (Brand & Perrimon, 1993), resulting in eye‐specific expression. Cryo‐electron microscopy showed degeneration of the individual hexagonal ommatidia from flies expressing the SCA3‐Q78, which was not observed in wild‐type flies (Fig. S2, Supporting information). This degeneration was also readily visible by light microscopy and was used to score eye degeneration.

**Figure 5:**
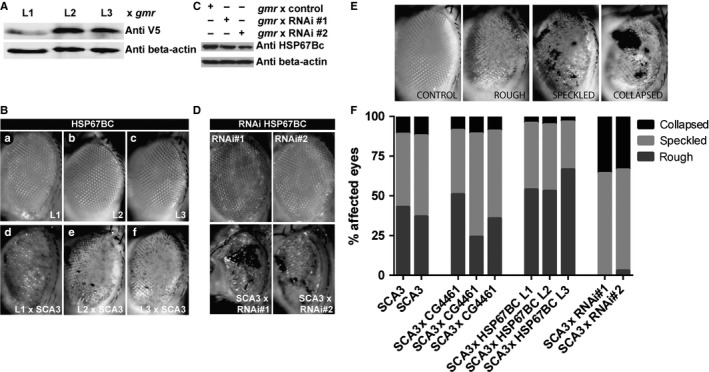
Effect of overexpression of the *Drosophila melanogaster* small heat‐shock proteins (HSPs) *in vivo* on reducing polyQ toxicity. (A) Flies transgenic for *UAS*‐V5‐HSP67BC (L1‐L3) were crossed with the *gmr*‐GAL4 driver line to verify transgene expression. Protein levels of head tissue are depicted. (B) ataxin‐3 flies (SCA3; gmr*‐GAL4 UAS‐SCA3trQ78/+*) were crossed with *UAS*‐V5‐HSP67BC (L1‐L3) transgenic lines (a–c), and the effects on eye degeneration (d–f) were analyzed by light microscopy. (C) Knockdown of endogenous HSP67BC was verified by crossing two *UAS*‐HSP67BC‐RNAi lines (RNAi#1, RNAi#2) with the *gmr‐GAL4*‐driver line. Protein levels of head tissue are depicted. (D) The *UAS*‐HSP67BC‐RNAi lines (RNAi#1, RNAi#2) were crossed with the ataxin‐3 fly line (SCA3; gmr*‐GAL4 UAS‐SCA3trQ78/+)*, and the effects on eye degeneration are depicted. (E) Degenerative eye phenotype was scored by light microscopy into three categories: rough, speckled, and collapsed. For this, the genotype *gmr‐GAL4 UAS‐SCA3trQ78/+* was used. Genotype *UAS‐SCA3trQ78/+* was used to generate the control image. (F) Quantification of degenerative eye phenotype from the crosses given in B and D as percentage of affected eyes is given. CG4461 overexpression had no effect on SCA3 eye phenotype. HSP67BC overexpression significantly reduced the collapsed eye phenotype (*t*‐test *P* < 0.008). HSP67BC knockdown reduced the rough phenotype (*t*‐test, *P* < 0.008) and increased speckled and collapsed phenotypes (*t*‐test, *P* < 0.03 and *P* < 0.008, respectively). Total numbers of eyes scored: SCA3, 370; CG4461 overexpression, 110; HSP67BC overexpression, 213; HSP67BC RNAi, 47. Genotypes used for the crosses: SCA3; gmr*‐GAL4 UAS‐SCA3trQ78/+,* CG4461; *UAS‐V5‐CG4461*. HSP67BC L1‐L3; *UAS‐V5‐HSP67BC*, RNAi#1 and RNAi#2*; UAS‐HSP67BC‐RNAi* (VDRC transformant lines ID 26416 and 26417).

Three independent transgenic lines expressing the V5‐tagged HSP67BC (Fig. 5A) showed eye degeneration upon crossing with the SCA3 fly model (Fig. 5A). Downregulation of HSP67BC in two independent RNAi lines (see Experimental procedures and Table S1, Supporting information) led to a small, but significant decrease in endogenous HSP67BC protein levels (Fig. 5C) and aggravated eye degeneration compared to HSP67BC overexpression (Fig. 5D).

For quantification, eye degeneration was scored into three readily visible degeneration patterns with increasing degenerative phenotypes: rough, speckled, and collapsed (Fig. 5E). As a negative control to further exclude nonspecific effects, we used three lines overexpressing V5‐tagged CG4461, one of the small HSPs that is not active in either refolding luciferase or polyQ aggregation (Figs 2 and 4A). In line with the observation that HSP67BC can suppress the aggregation of polyglutamine containing proteins in cells (Fig. 4A), overexpression of HSP67BC in the SCA3 model significantly reduced the collapsed eye phenotype in all three individual lines tested (Fig. 5F). Downregulation of endogenous HSP67BC in two independent RNAi lines showed both a significant reduction in the rough phenotype and a significant increase in the speckled and collapsed phenotypes, consistent with the anti‐aggregation activity identified in Fig. 4A. The current data are consistent with our previous findings that HSP67BC, the functional ortholog of the human HSPB8, is able to modulate eye degeneration caused by expression of proteins containing expanded polyglutamine repeats (Carra *et al*., 2010).

### Linking chaperone activities to longevity

**Figure 6:**
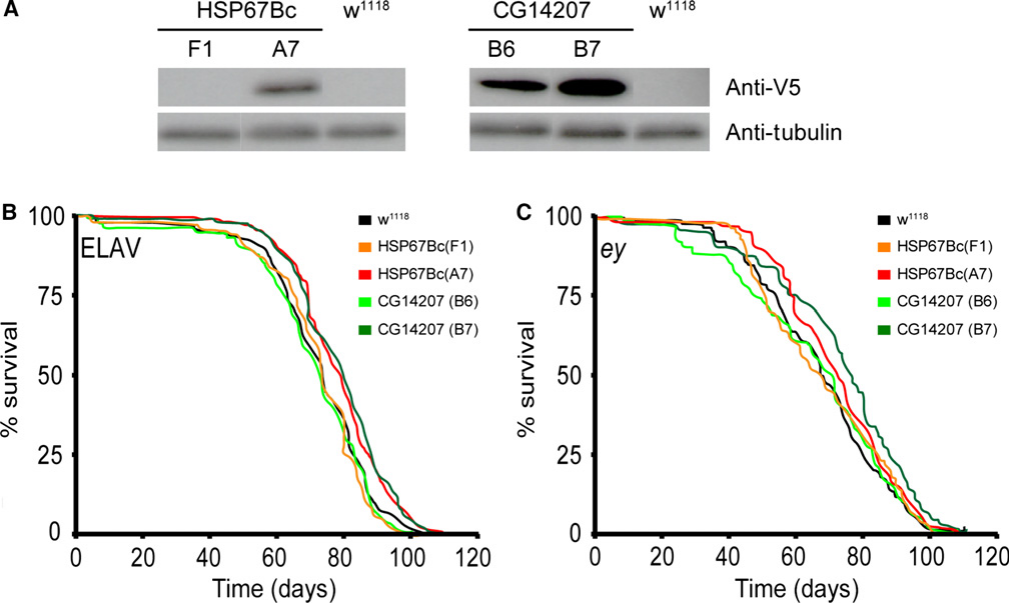
Effect of overexpression of the *Drosophila melanogaster* small heat‐shock proteins (HSP) *in vivo* on lifespan. Large cohorts of male flies were analyzed for their lifespan. Overexpression of each sHSP studied (using the *ey*‐GAL4‐driver) was confirmed by Western blot analysis (A). Isogenic controls (*w* ^*1118*^ lines) or transgene expression lines (*UAS*‐HSP67Bc(F1), *UAS*‐HSP67Bc(A7), *UAS*‐CG14207(B6), and *UAS*‐CG14207(B7)) were crossed with *elav‐GAL4* driver (B) or *ey‐GAL4* driver (C) lines, and survival curves of the progeny expressing the transgenes were plotted using Kaplan–Meier and statistically analyzed using log‐rank (Mantel–Cox). As a control, lifespan of the progeny of driver lines crossed with the isogenic controls (*w* ^*1118*^) was determined. Significant differences between survival curves were observed between isogenic control (median survival = 74 days) and HSP67Bc(A7) (median survival = 77.5 days) (*P* < 0.001), and between the isogenic control (median survival = 74 days) and CG14207(B7) (median survival = 80 days) (*P* < 0.0001) driven by *elav‐GAL4*, and significant differences between survival curves were observed between isogenic control (median survival = 68 days) and HSP67Bc(A7) (median survival = 72 days) (*P* < 0.003), and between the isogenic control (median survival = 68 days) and CG14207(B7) (median survival = 76 days) (*P* < 0.0001) driven by *ey‐GAL4*. The total number of flies analyzed was 647 for *w* ^*1118*^, 607 for F1, 522 for A7, 479 for B6, 484 for B7 (panel B), 651 for w1118, 553 for F1, 486 for A7, 484 for B6, and 435 for B7 (panel C).

Finally, we wondered whether the separate activities of HSP67BC and CG14207 on protein stress could have implications during aging. Previous studies have shown that overexpression of HSP22, HSP23, HSP26, and HSP27 can extend lifespan in flies (Aigaki *et al*., 2002; Morrow *et al*., 2004; Wang *et al*., 2004). While for the mitochondrial HSP22 these effects were attributed to improved quality control within mitochondria and likely involve a reduction in oxidative stress (Morrow *et al*., 2004), the effects of the HSP23, HSP26, and HSP27 can be associated with an improved protein homeostasis in the cytosol and/or nucleus of the cells. Here, we showed that the latter 3 sHSPs have intermediate activities both in assisting refolding of stress‐denatured substrates and in preventing aggregation of disease‐associated misfolded proteins. To uncover whether either one of these activities can also support longevity, we determined the lifespan of male flies overexpressing CG14207 (strong refolder) and HSP67BC (strong anti‐aggregation) using *elav‐GAL4* or *ey‐GAL4* promoters. *Elav‐GAL4* drives expression in the central and peripheral nervous systems (Robinow & White, 1991). The *ey‐GAL4* driver, which is traditionally used for eye‐specific expression, also induces expression in the rest of the body (flyatlas http://www.flyatlas.org). To properly evaluate the effects of single gene overexpression on lifespan, we backcrossed all lines over *w* ^*1118*^ males for six generations (Burnett *et al*., 2011). This led to loss of expression of the transgenes in some lines (Fig. 6A, HSP67Bc‐F1), which served as an additional internal control line for which the lifespan was unaltered compared to the isogenic controls crossed with the driver lines (Fig. 6B,C). Overexpression of HSP67BC (line A7) resulted in a significant, albeit small increase in longevity when expression was driven by either *elav‐GAL4* or *ey‐GAL4* (Fig. 6B,C). For CG14207, only the line showing the highest expression (Fig. 6A, CG14207‐B7) showed a significant increase in lifespan when expression was driven by *elav‐GAL4* or *ey‐GAL4*. For the other line (CG14207‐B6), the survival curve, however, was unaltered compared to the respective isogenic controls (Fig. 6B,C). Although overexpression of CG14207 and HSP67BC only showed a mild increase in lifespan, our results demonstrate that functionally diverse small HSP members can promote longevity.

## Discussion

To unravel the functional divergence of small HSPs and their effects on organismal health, we compared the *Drosophila* small HSPs for heat inducibility their ability to assist in protein refolding and ability to prevent polyQ aggregation. Intriguingly, we identified two small HSPs, which either exclusively supported refolding (CG14207) or prevented aggregation (HSP67BC). CG14207 was the only small HSP that was not heat‐inducible and in addition depended on HSP70 for its refolding activity. HSP67BC, on the other hand, was clearly heat‐inducible and showed the strongest, but HSP70‐independent, activity in preventing polyQ aggregation, while its overexpression did not increase the cellular ability to refold heat‐denatured luciferase. In addition, the overexpression of both single small HSPs resulted in increased lifespan compared to their isogenic controls.

### Classical HSP70 activity and anti‐aggregation

Chaperone‐like actions of small HSPs have generally been suggested to depend on HSP70 activity. In protein refolding assays *in vitro* (Horwitz, 1992; Jakob *et al*., 1993), aggregation prevention by the ATP‐independent small HSPs has been shown to occur independent of the ATP‐dependent HSP70 machinery, but for efficient refolding, substrate transfer to the HSP70 machine is required (Lee & Vierling, 2000; Mogk *et al*., 2003). Also, in living cells, refolding assistance by HSPB1 was found to require a functional HSP70 machinery (Bryantsev *et al*., 2007), and in this study, a similar scenario was found to be true for the best refolding stimulating *D. melanogaster* small HSP, CG14207. Strikingly, CG14207 was ineffective in preventing polyQ aggregation. Inversely, the best protector against polyQ aggregation, HSP67BC, acted independent of the HSP70 machine and was ineffective in the luciferase refolding assay. We found a similar pattern for members of the human family of small HSP proteins as well, where the efficacy to assist in HSP70‐dependent refolding was almost inversely related to the ability to prevent polyQ aggregation (Vos *et al*., 2010). One can envision that transfer of nonfoldable substrates to HSP70 would result in a fatuous ATP‐driven substrate binding and release, which may slightly delay aggregation but not prevent it. Indeed, in cells overexpression of HSP70s only marginally affects polyQ aggregation (Chai *et al*., 1999; Rujano *et al*., 2007), whereas *in vivo* HSP70‐mediated rescue of polyQ toxicity only occurs in the presence of semi‐soluble nuclear polyQ aggregates (Warrick *et al*., 1999; Chan *et al*., 2000; Cummings *et al*., 2001). Thus, the different small HSPs with different affinities to substrates and HSP70s may have evolved to serve adequate processing of a broad spectrum of clients. The low HSP70 and high substrate affinity may serve to prevent aggregation of unfoldable substrates and provide a longer time window for ubiquitination and normal proteasomal turnover and/or autophagic processing.

### Inducing longevity

Several screens have been performed as an attempt to elucidate the molecular events underlying aging (Zou *et al*., 2000; Pletcher *et al*., 2002). This has revealed changed expression of several genes involved in many cellular pathways, including members of HSP families. Instead of looking at physiologically induced aging effects on gene expression, we tried to determine whether individual molecular functions of small HSPs, respectively HSP70‐dependent assistance of (re)folding reactions (CG14207) or HSP70‐independent prevention of polyQ aggregation (HSP67BC), can contribute to an enhanced lifespan in line with the theory that overall protein homeostasis is important for healthy aging (Balch *et al*., 2008) and that a multitude of molecular chaperones of which the expression is regulated via HSF‐1, a transcriptional regulator of stress‐inducible gene expression, are vital in both the protection against protein folding diseases and aging (Walker *et al*., 2001; Morley & Morimoto, 2004; Cohen *et al*., 2006). In line, previous data had already demonstrated that the HSP22, the mitochondrial *D. melanogaster* small HSP family member, can induce longevity (Morrow *et al*., 2004). We now show that two other small HSP members that have activities on only one of the two substrates investigated here, one related to acute stress (CG14207) and one to chronic stress (HSP67Bc), also are capable of extending lifespan in *Drosophila*, demonstrating that increased levels of functionally diverse small HSPs can promote longevity *in vivo* by different protective activities that yet both contribute to an improved protein homeostasis. Notably, a recent extensive study on proteome remodeling and aggregation in aging *C. elegans* (Walther *et al*., 2015) showed that among all chaperones, particularly small HSPs were associated with the increase in protein homeostasis in long‐lived daf‐2 mutant worms, consistent with the view that small HSPs play a pivotal role in the lifespan‐prolonging effect of the insulin/insulin‐like growth factor‐1 signaling pathway.

## Experimental procedures

### Organisms and growth conditions

For cloning purposes and plasmid isolation, *Escherichia coli* DH5α (Gibco‐BRL, Gaithersburg, MD, USA) was used and grown at 37 °C in LB medium (1% bacto‐tryptone, 0.5% yeast extract, 0.5% NaCl), supplemented with the appropriate antibiotics when required.

*Drosophila* Schneider's S2 cells were cultured in Schneider's *Drosophila* medium (GIBCO, Paisley, UK) supplemented with 10% heat‐inactivated fetal bovine serum (Greiner, Alphen aan den Rijn, the Netherlands), 100 units per mL penicillin, and 100 g mL^−1^ streptomycin in T25 flasks at 25 °C. For exponential cell growth, cell density was kept between 3 × 10^5^ and 3 × 10^6^ cells mL^−1^.

Fly stocks (Table S1) were maintained at 22 °C according to standard protocols. *GAL4* driver stocks were obtained from the Bloomington Stock Center (Indiana University, USA) (Table S1). The fly stock bearing *gmr‐GAL4 UAS‐SCA3Q78* used for the eye‐degeneration screen was generously provided by N. Bonini (University of Pennsylvania, USA) and maintained at 25 °C. Transgenic lines were generated by Genetic Services Inc. (Sudbury, MA, USA) by injection of the pUAS vector, harboring V5‐HSP67BC, V5‐CG4461, or V5‐CG14207, into the *w* ^*1118*^ genetic background. For longevity analyses, HSP‐expressing males were crossed with *w* ^*1118*^ virgins, and next, the female offspring was backcrossed for six generations with male *w* ^*1118*^ to generate isogenic controls. We used the same procedure for the balancer flies. Finally, we crossed the balancers back into the HSP flies. Two independent RNAi lines (transformant ID 26416 (#1) and 26417 (#2)), to downregulate endogenous HSP67BC protein levels, were obtained from the VDRC stock collection (for details, see Table S1).

### Molecular techniques, bioinformatics, and plasmid generation

Oligonucleotide primers (Biolegio, Nijmegen, the Netherlands) and plasmids used in this study are listed in Tables S2–S5 (Supporting information). Standard recombinant DNA techniques were carried out essentially as described by Sambrook *et al*. (1989). Restriction enzymes were used according to the manufacturers' instructions (Invitrogen, Breda, the Netherlands; and New England Biolabs, Ipswich, MA, USA). Vent DNA polymerase (New England Biolabs) was used for preparative polymerase chain reactions. DNA sequencing reactions were carried out by ServiceXS (Leiden, the Netherlands). Blast algorithms (McGinnis & Madden, 2004) were used to screen databases at the National Center for Biotechnology Information (NCBI) (Bethesda, MD, USA).

*Drosophila* small heat‐shock protein sequences were retrieved from the NCBI database using the *D. melanogaster* HSP27 sequence as input for a protein BLAST search. Results were analyzed by clustalx (Larkin *et al*., 2007) and visualized by treeview (Page, 1996). A total of eleven small HSPs were found to be present in the *D. melanogaster* genome, most of which are located at position 67B on the third chromosome (Fig. S1A, Supporting information). Nearest‐neighbor analysis shows three main groups within the *D. melanogaster* small HSP family (Fig. S1B). Group A represents the most studied classical small HSPs: HSP22, HSP23, HSP26, and HSP27. Group B consists of L(2)EFL, CG4461, CG7409, and CG14207, while group C contains HSP67BC and CG13133. The *Drosophila* HSP plasmid library (Fig. 1C) was generated using either cDNA originating from heat‐shocked flies or cDNA from the Gold cDNA library (Bloomington, Indiana University). Primers used for isolation and amplification of individual small HSPs using are listed in Table S2. All PCR products were cloned into the pAc5.1‐V5 plasmid and sequence verified. The pAc5.1‐V5 was generated by annealing two oligonucleotides forming a KpnI overhang at the 5′ end (Table S2). This fragment was ligated into KpnI‐EcoRV‐digested pAc5.1.

The L4440 RNAi feeding vector (Fire Laboratory), containing two opposing T7 sequences, was modified for T/A‐cloning as follows. To remove unneeded nucleotides between both T7 sequences, L4440 was digested with BglII and XhoI. Annealed oligonucleotides (Table S2), designed to provide BglII and XhoI overhangs and two internal XcmI sites, were ligated into digested L4440 leading to L4440‐T/A. Upon digestion with XcmI, this plasmid contains two T‐overhangs, allowing annealing of Taq DNA polymerase‐amplified DNA fragments (A‐overhangs). To generate the dsRNA‐template library, a specific part of the HSP70 and HSC70 genes was amplified using primers listed in Table S3 using Taq polymerase. Subsequently, the A‐tailed PCR products were ligated into the XcmI‐digested L4440‐T/A plasmid.

### DNA transfection

Schneider's S2 cells were transfected using either the CaCl_2_ method or Effectene (Qiagen, Venlo, the Netherlands). Transfection using the CaCl_2_ was performed as follows. A total of 2.5 μg of plasmid DNA was mixed with 10 μL 2.5 m CaCl_2_, the volume was adjusted to 100 μL with 0.1 × TE (pH 7.6), and this was added dropwise to 100 μL 2 ×  HEPES buffer (280 mm NaCl, 1.5 mm Na_2_HPO_4_ 2H_2_O, 50 mm HEPES, pH 7.05) while vortexing. Precipitates were allowed to form for 30 min, and then, the solution was added to 1 × 10^6^ cells in a 35‐mm dish. Transfection using Effectene was performed according to the manufacturer's instructions using 0.6 μg of plasmid DNA in combination with 5 μL Effectene.

### RNA interference

RNA interference was performed as described previously (Clemens *et al*., 2000). In short, DNA fragments of variable length coding for specific parts of the target genes (Table S3) were amplified using Taq DNA polymerase. These fragments were cloned into XcmI‐digested L4440‐T/A vector. Using T7‐specific primers, the T7‐flanked target sequence was re‐amplified. Subsequently, dsRNA was generated using the MEGASCRIPT T7 transcription kit (Ambion, Austin, TX, USA). S2 cells were diluted to a final concentration of 1x10^6^ cells/mL in *Drosophila* serum‐free medium (GIBCO, Paisley, UK). dsRNA was added to the serum‐free medium and incubated for 1 h at 25 °C, followed by the addition of 2 mL complete Schneider's medium. Part of an intron sequence of the human MAZ gene was used as mock dsRNA. One day after dsRNA transfection, cells were transfected with plasmid DNA. Two days later, cells were subjected to either the luciferase refolding assay or the filter trap assay.

### Quantitative PCR

Exponentially growing Schneider's S2 cells were resuspended in complete Schneider's *Drosophila* medium to a final concentration of 1 × 10^6^ cells per mL. A total of 5 mL of cell suspension was heat‐shocked for the indicated temperature and time points using a precision water bath. Total RNA was isolated using the Invisorb Spin Cell RNA mini kit (Westburg, Leusden, the Netherlands). First‐strand cDNA was generated using M‐MLV reverse transcriptase (Invitrogen) using oligo(dT)_18_ primers (Biolegio, Nijmegen, the Netherlands). Relative changes in transcript level were determined using the iCycler (Bio‐Rad, Hercules, CA, USA) in combination with SYBR Green Supermix (Bio‐Rad, Veenendaal, the Netherlands). Calculations were performed using the comparative C_T_ method according to User Bulletin 2 (Applied Biosystems, Foster City, CA, USA; Livak & Schmittgen, 2001). The PCR efficiencies for all primer pairs (Table S5) were between 85% and 100%. Fold induction was adjusted using RpL32 transcript levels as a standard.

### Luciferase refolding assay

The luciferase refolding assay (Michels *et al*., 1995) was adapted for Schneider's S2 cells as follows. S2 cells were transfected with the pAc5.1‐Luc plasmid, coding for firefly luciferase together with different heat‐shock‐protein‐coding plasmids in a 1:9 ratio. After two days, cells were resuspended in complete Schneider's *Drosophila* medium containing cycloheximide (2 mg/100 mL). The cell suspension was divided into 400‐μL portions in 1.5‐mL centrifuge tubes. Tubes were placed into custom‐made acrylic glass racks (50 positions), which allowed continuous water flow around the tubes when placed in a water bath. After a heat treatment of 30 min at 38 °C, the tubes were cooled down rapidly by placing them in a 25 °C water bath followed by incubation in a 25 °C incubator. Cell lyses and luciferase measurements were performed as described (Michels *et al*., 1995). The experiments were performed and measured in triplicate.

### Biochemical techniques

SDS‐PAGE and Western blotting were performed by established procedures. Primary antibodies used were monoclonal anti‐EGFP (Clontech, Saint‐Germain‐en‐Laye, France) and monoclonal anti‐V5 (Invitrogen) according to the manufacturers' instruction. The filter trap binding assay was performed as described previously (Carra *et al*., 2005).

### Antibody preparation

The rabbit polyclonal antibody anti‐HSP67BC was raised against the C‐terminal peptide CHKEAGPAASASEPEAK of *Drosophila melanogaster* HSP67BC coupled to the keyhole limpet hemocyanin.

### Lifespan analysis

For lifespan analysis, male flies were selected and maintained at noncrowding conditions at 22 °C. Dead flies were counted three times per week followed by transfer to fresh media. Lifespan curves were visualized in Kaplan–Meier plots and analyzed using a log‐rank (Mantel–Cox) test for significance.

## Funding info

This work was supported by Senter (Innovatiegerichte Onderzoeksprogramma genomics Grant IGE03018 to H.H.K.).

## Conflict of interest

None declared.

## Supporting information


**Fig. S1** The *Drosophila* family of small heat‐shock proteins.

**Fig. S2** ataxin‐3 fly model for polyQ‐related eye degeneration.

**Table S1** Drosophila lines used in this study.

**Table S2** Primers used for molecular cloning.

**Table S3** Primers used for the generation of dsRNA and specificities of the dsRNA sequences.

**Table S4** Plasmids used in this study.

**Table S5** Primers used for qPCR.
