# Supplementary material for: Specific protein homeostatic functions of small heat‐shock proteins increase lifespan
Source: Aging Cell. 2015 Dec 25;15(2):217–26. doi: 10.1111/acel.12422 (PMC4783350; doi:10.1111/acel.12422)

A

The Drosophila small HSP family				
**CG number**	**Gene name**	**Chromosome**	**Locus**	**GeneID**
CG4460	*HSP22*	3L	67B	3772576
CG4463	*HSP23*	3L	67B	39077
CG4183	*HSP26*	3L	67B	39075
CG4466	*HSP27*	3L	67B	39078
CG4167	*HSP67BA*	3L	67B	39076
CG4190	*HSP67BC*	3L	67B	39071
CG4533	*L(2)EFL*	2R	59F	37744
CG4461	-	3L	67B	39074
CG7409	-	3L	66A	38870
CG13133	-	2L	31A	34342
CG14207	-	X	18D	32955

B

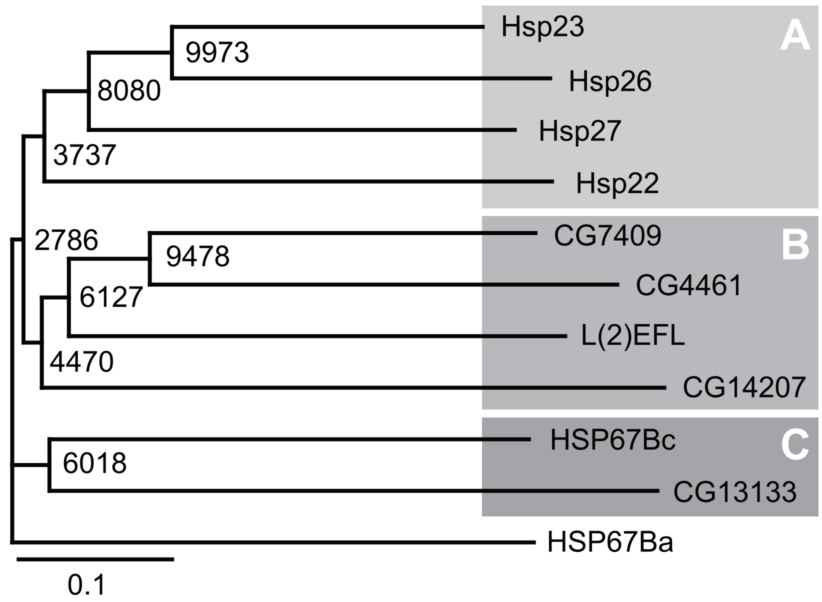

C

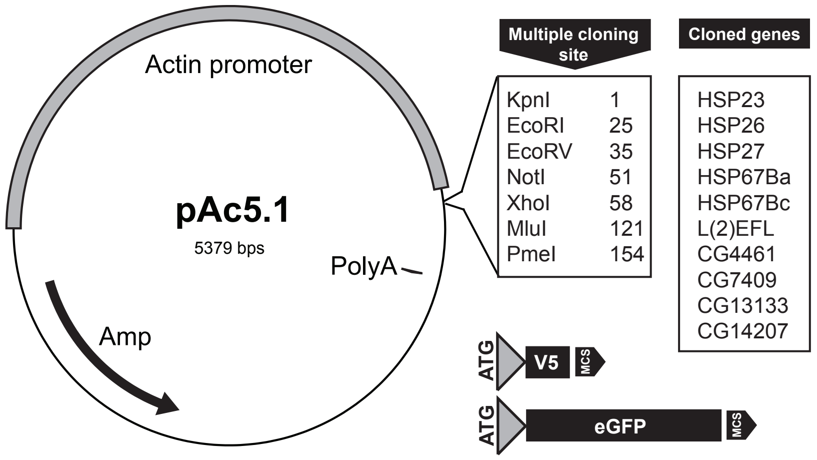

Supplement: Supplementary file 1 — Fig. S1 The Drosophila family of small heat‐shock proteins. [file ACEL-15-217-s001.pdf]
