# Supplementary figures and images for: Specific protein homeostatic functions of small heat‐shock proteins increase lifespan
Source: Aging Cell. 2015 Dec 25;15(2):217–26. doi: 10.1111/acel.12422 (PMC4783350; doi:10.1111/acel.12422)

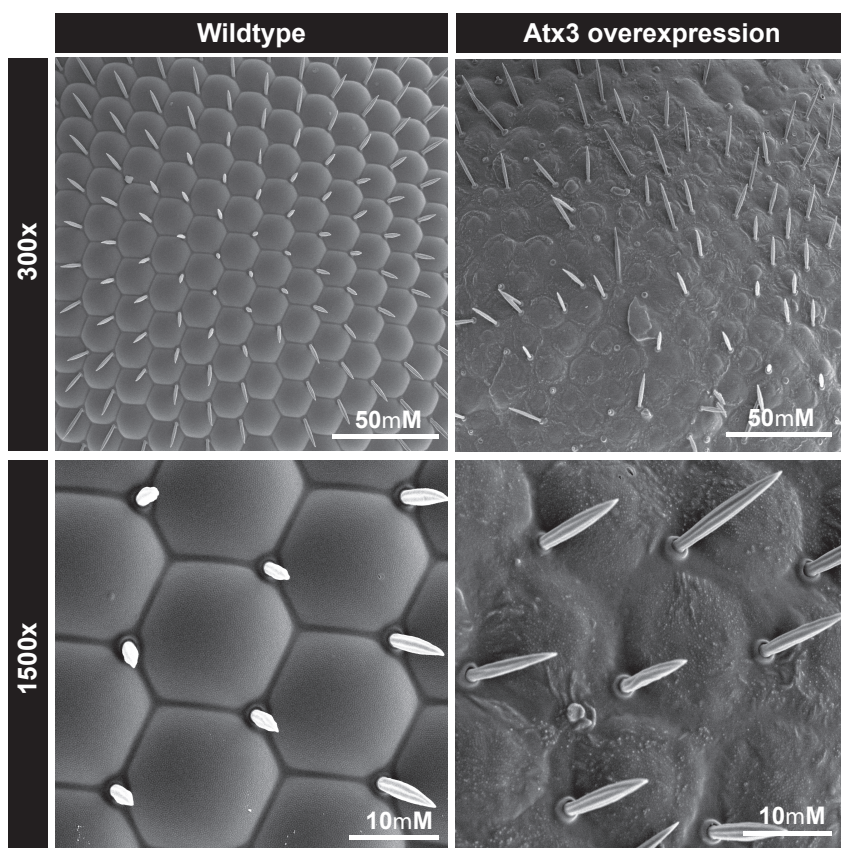

Supplement: Supplementary file 2 — Fig. S2 ataxin‐3 fly model for polyQ‐related eye degeneration. [file ACEL-15-217-s002.pdf]
