# Supplementary material for: Specific protein homeostatic functions of small heat‐shock proteins increase lifespan
Source: Aging Cell. 2015 Dec 25;15(2):217–26. doi: 10.1111/acel.12422 (PMC4783350; doi:10.1111/acel.12422)
Supplement: Supplementary file 3 — Table S1 Drosophila lines used in this study. [file ACEL-15-217-s003.pdf]

Table S1 Drosophila lines used in this study

Name	Genotype and characteristics	Reference
W1118	Wildtype strain	Genetic Services
W1118-TM3	TM3, Ser1, Sb1	This study
SCA3trQ78	GMR-GAL4 UAS-SCA3trQ78/In(2LR), Cy	N. Bonini
HSP67BC-L1	UAS-V5-CG4190-A7/TM3, Ser1, Sb1	This study
HSP67BC-L2	UAS-V5-CG4190-F1/TM3, Ser1, Sb1	This study
HSP67BC-L3	UAS-V5-CG4190-F2/TM3, Ser1, Sb1	This study
CG4461-L1	UAS-V5-CG4461-L04/In(2LR)O,Cy	This study
CG4461-L2	UAS-V5-CG4461-L05/In(2LR)O,Cy	This study
CG4461-L3	UAS-V5-CG4461-L07/TM3, Ser1, Sb1	This study
CG4461-L4	UAS-V5-CG4461-L10/In(2LR)O,Cy	This study
CG14207-L3	UAS-V5-CG14207-B6/TM3, Ser1, Sb1	This study
CG14207-L4	UAS-V5-CG14207-B7/TM3, Ser1, Sb1	This study
HSP67BC RNAi 1	UAS-HSP67BC-RNAi / VDRC Transformant ID 26416	VDRC
HSP67BC RNAi 2	UAS-HSP67BC-RNAi / VDRC Transformant ID 26417	VDRC
actin-GAL4	y[1] w[*]; P{w[+mC]=Act5C-GAL4}25FO1/CyO, y[+] / 4414 / ubiquitous expression	Bloomington
elav-GAL4	w^{*}; P{GAL4-elav.L}2/CyO / 8765 / high expression in CNS	Bloomington
ey-GAL4	y^{1} w^{1118}; P{ey1x-GAL4.Exel}2 / 8228 / moderate ubiquitous expression	Bloomington
gmr-GAL4	w[*]; P{w[+mC]=GAL4-ninaE.GMR}12 / 1104 / expression in eye tissue	Bloomington
