# Supplementary material for: Specific protein homeostatic functions of small heat‐shock proteins increase lifespan
Source: Aging Cell. 2015 Dec 25;15(2):217–26. doi: 10.1111/acel.12422 (PMC4783350; doi:10.1111/acel.12422)
Supplement: Supplementary file 4 — Table S2 Primers used for molecular cloning. [file ACEL-15-217-s004.pdf]

Table S2 Primers used for molecular cloning

Gene family	Primer name	Primer sequence (5'-3')
**sHSP**	HSP23 F	ACCCCTCGAGCACCATGGCAAATATTCCATTGTTGT
	HSP23 R	ACCCACGCGTAAATAATGCAGGGCATCTCT
	HSP26 F	ACCGCTCGAGGACCATGTCGCTATCTACTCTGCTTTC
	HSP26 R	ACCCACGCGTGGCTCCTTTACTTGTCCTTG
	HSP27 F	ACCAGCTCGAGACCATGTCAATTATACCACTGCTGCAC
	HSP27 R	ACCCACGCGTTTCTCGTCTTCTTCATCTCCTAGTC
	L(2)EFL F	ACAACTCGAGAACCATGTCCGTAGTGCCACTGAT
	L(2)EFL R	ACCCACGCGTACTAACACTGAAGGCGGTACA
	HSP67Ba F	ACCACTCGAGGACCATGTCGCTGATACCGTTCATAC
	HSP67Ba R	ACCCACGCGTTCACTTGGCCAGTTCCTCGGTTT
	HSP67Bc F	ACCCCTCGAGCACCATGCCAGATATTCCCTTTGT
	HSP67Bc R	ACCTACGCGTTCACTTGGCTTCTGGCTCTG
	CG4461 F	ACGCCTCGAGAGAAATGTCTCTGGTGCCTACC
	CG4461 R	ACCGACGCGTGACACATGGTCCACTATTTCC
	CG7409 F	ACAACTCGAGAACCATGGCTCTTGTCCCGGCTAC
	CG7409 R	ACCCACGCGTCTGTGATTAGTTCTTGATGC
	CG13133 F	AAACCTCGAGAACCATGCCCATCCACTGGGACTG
	CG13133 R	AACCACGCGTTCATTTGTCCATCGCCGTGCCAGCAG
	CG14207 F	AACCCTCGAGAACCATGGCCGAGGCTAACAAGAGG
	CG14207 F	ACCCACGCGTACTGGATAGCTGGACAACTGGA
**HSP40**	MRJ F	ACCCGATATCACCAACATGGTTGACTACTAT
	MRJ R	ACAAACGCGTGCTATTGAAGGGAGCCCATC
**HSP70**	DmHSP70Aa	GCGCGATATCACCATGCCTGCTATTGGAATCGATC
	DmHSP70Aa	GCAGCTCGAGTTAGACTCTTTGGCCTTAGTCG
	HSC70-2	ACCAGATATCACCATGGGTAAAATTCCGGCCATCGGCATCG
	HSC70-2	ACCAGCGGCCGCGGTCGGTGGATCGTACTATC
	HSC70-4	ACAAGCGGCCGCAAGATGTCTAAAGCTCCTGCTGTT
	HSC70-4	ACAAACGCGTATGGTTTAGTCGACCTCCTC
**Modifications**		
**L4440-XcmI**	XcmI-F stuf	TCGACCATGGTTTTGTTGGCCATGTTATCCATGG
	XcmI-R stuf	GATCCCATGGATAACATGGCCAACAAAACCATGG
**pAc5.1-V5**	V5 stuffer F	AGTACTACCATGGGTAAGCCTATCCCTAACCCTCTCCTCGGT CTCGATTCTACGGAT
	V5 stuffer R	ATCCGTAGAATCGAGACCGAGGAGAGGGTTAGGGATAGGCT TACCCATGGTAGTACTGTAC
