# Supplementary material for: Specific protein homeostatic functions of small heat‐shock proteins increase lifespan
Source: Aging Cell. 2015 Dec 25;15(2):217–26. doi: 10.1111/acel.12422 (PMC4783350; doi:10.1111/acel.12422)
Supplement: Supplementary file 5 — Table S3 Primers used for the generation of dsRNA and specificities of the dsRNA sequences. [file ACEL-15-217-s005.pdf]

Table S3 Primers used for the generation of dsRNA and specificities of the dsRNA sequences

Primer name or fly	Primer sequence (5'-3')	Template	Region	On-target siRNA's	HSP off-target Gene	Mismatch 0	Mismatch 1	Mismatch 2
HsMAZ-F	GTGGCGTCTAGATTCCTACAAG	NC_000016 REGION:	2310-3028	0	none			
HsMAZ-F	AGGTATGCTGCCGTGGTGAA	29725356..29730005						
RNAi Hsp70x F	AAGCATCGCCAGCGAATAAC	CG31366-RA	2088-2408	303*	HSP68	3	22	14
RNAi Hsp70x R	TCCAGAGTAGCCTCCAAATC							
RNAi Hsc70-2 F	CCTTCGACGTCTCCGTACTG	CG7756-PA	807-1193	369	HSP70	1	3	9
RNAi Hsc70-2 R	GAATGGCCTTCTTGTCCATC				HSC70-4	0	7	27
RNAi Hsc70-4 F	TGAACGTGCTGCGTATCATC	CG4264-PA	655-1059	387	HSC70-3	5	18	29
RNAi Hsc70-4 R	AACGGGCACGAGTAATCGAG				HSP70	4	26	37
**Transgenic lines**								
UAS-HSP67BC-RNAi	Obtained from VDRC			322	HSP27	0	1	6

* This pool of siRNA's targets both the HSP70A and HSP70B members.
On-target and off-target siRNA's were determined using dsCheck located at http://dscheck.rnai.jp
