# Supplementary material for: Specific protein homeostatic functions of small heat‐shock proteins increase lifespan
Source: Aging Cell. 2015 Dec 25;15(2):217–26. doi: 10.1111/acel.12422 (PMC4783350; doi:10.1111/acel.12422)
Supplement: Supplementary file 6 — Table S4 Plasmids used in this study. [file ACEL-15-217-s006.pdf]

**Table S4** Plasmids used in this study

| | Plasmid name | Characteristics | Reference |
|---|---|---|---|
| **Cloning** | pAc5.1 | *Dm* Actin promoter | Invitrogen |
| | pAc5.1-V5 | *Dm* Actin promoter / N-terminal V5-tag | This study |
| | pUAST-V5 | Transformation vector / N-terminal V5-tag | This study |
| | L4440-T/A | Adapted RNAi feeding vector (Fire laboratory) | This study |
| **General** | pAc5.1-EGFP | Enhanced Green Fluorescent Protein | This study |
| | pAc5.1-Luc-EGFP | EGFP tagged luciferase / cloned from pGL3 (Promega) | This study |
| | pAc5.1-HttQ119-EGFP | EGFP tagged Huntingtin exon 1 with 119 glutamines | This study |
| **sHSP** | pAc5.1-V5-HSP23 | *Dm* HSP23 / GeneID: 39077 | This study |
| | pAc5.1-V5-HSP26 | *Dm* HSP26 / GeneID: 39075 | This study |
| | pAc5.1-V5-HSP27 | *Dm* HSP27 / GeneID: 39078 | This study |
| | pAc5.1-V5-HSP67BA | *Dm* HSP67BA / GeneID: 39076 | This study |
| | pAc5.1-V5-HSP67BC | *Dm* HSP67BC / GeneID: 39071 | This study |
| | pAc5.1-V5-L(2)EFL | *Dm* L(2)EFL / GeneID: 37744 | This study |
| | pAc5.1-V5-CG4461 | *Dm* CG4461 / GeneID: 39074 | This study |
| | pAc5.1-V5-CG7409 | *Dm* CG7409 / GeneID: 38870 | This study |
| | pAc5.1-V5-CG13133 | *Dm* CG13133 / GeneID: 34342 | This study |
| | pAc5.1-V5-CG14207 | *Dm* CG14207 / GeneID: 32955 | This study |
| **HSP40** | pAc5.1-V5-MRJ | *Dm* MRJ / GeneID: 36797 | This study |
| **HSP70** | pAc5.1-V5-HSP70AA | *Dm* HSP70AA / GeneID: 48581 | This study |
| | pAc5.1-V5-HSC70-2 | *Dm* HSC70-2 / GeneID: 41609 | This study |
| | pAc5.1-V5-HSC70-4 | *Dm* HSC70-4 / GeneID: 41840 | This study |
