# Supplementary material for: Specific protein homeostatic functions of small heat‐shock proteins increase lifespan
Source: Aging Cell. 2015 Dec 25;15(2):217–26. doi: 10.1111/acel.12422 (PMC4783350; doi:10.1111/acel.12422)
Supplement: Supplementary file 7 — Table S5 Primers used for qPCR. [file ACEL-15-217-s007.pdf]

Table S5 Primers used for qPCR

Gene family	Primer name	Primer sequence (5'-3')
**sHSP**	qPCR HSP22 F	CTGTCAAGAGTATAAATAGCCACC
	qPCR HSP22 R	GTTGTCTGAGGTTTCAATCGT
	qPCR HSP23 F	CAATGGCAACGATAAGTAGAG
	qPCR HSP23 R	TGTGTATGAGTGAAGTGTCCT
	qPCR HSP26 F	CGCATCATTCAAATTCAGCA
	qPCR HSP26 R	ATGGCTCCTTTACTTGTCCT
	qPCR HSP27 F	CTAGACAGGGTTGTGAATAAAGAG
	qPCR HSP27 R	AAACCGAAGTCATCCTCCAG
	qPCR L(2)EFL F	GAAGTTCGAGGTCATTCTGG
	qPCR L(2)EFL R	CTTCTCCTCATGCTTTCCCT
	qPCR HSP67Ba F	CACTTCCACTTTAAATCCGACTG
	qPCR HSP67Ba R	TTTCCATCTCTGTCTCTTCCT
	qPCR HSP67Bc F	GGTTCCACCATTAGTTTCCA
	qPCR HSP67Bc R	CCTCCTTATGACCGTTTCCA
	qPCR CG4461 F	CCAGGGAAATCACAATCGTC
	qPCR CG4461R	CTGGATATGTCCGAGATTACCT
	qPCR CG7409 F	GATTGGGATTTGAACGACTGG
	qPCR CG7409 R	TCTCATAGGGCTTGAACAGG
	qPCR CG13133 F	GTGCACCACTTTCAGATCTC
	qPCR CG13133 R	CTTGTAGGATCGCGTAAACTC
	qPCR CG14207 F	TTAGCTCACCCTTGATCCAG
	qPCR CG14207 R	TTTGTGTCCGACTTCTCCTC
**HSP70**	qPCR HSP70Aa F	ACCTCAACCTATCCATCAACC
	qPCR HSP70Aa R	GTCTCAATTCCCAATGAAAGTG
	qPCR HSC70-4 F	CTGCTGTTGGTATTGATTTGG
	qPCR HSC70-4 R	CATAGGATGGAGTGGTACGA
	qPCR HSC70-5 F	CACTATTCCCACCAAGAAGTC
	qPCR HSC70-5 R	TTATTGTCGTTAGCCATCTCAC
**General**	qPCR RpL32 F	ACATGCTGCCCACCGGATTC
	qPCR RpL32 R	GCGATCTCGCCGCAGTAAAC
